# Pollutant Degrading Enzyme: Catalytic Mechanisms and Their Expanded Applications

**DOI:** 10.3390/molecules26164751

**Published:** 2021-08-06

**Authors:** Anming Xu, Xiaoxiao Zhang, Shilei Wu, Ning Xu, Yan Huang, Xin Yan, Jie Zhou, Zhongli Cui, Weiliang Dong

**Affiliations:** 1 State Key Laboratory of Materials-Oriented Chemical Engineering, College of Biotechnology and Pharmaceutical Engineering, Nanjing Tech University, Nanjing 211800, China; xu_anming@njtech.edu.cn (A.X.); x.x.zhang@njtech.edu.cn (X.Z.); wushilei_njtech@163.com (S.W.); xuning@hytc.edu.cn (N.X.); jayzhou@njtech.edu.cn (J.Z.); 2 Key Laboratory of Agricultural Environmental Microbiology, Ministry of Agriculture and Rural Affairs, College of Life Sciences, Nanjing Agricultural University, Nanjing 210095, China; huangyan@njau.edu.cn (Y.H.); yanxin@njau.edu.cn (X.Y.)

**Keywords:** microbial enzyme, catalytic mechanism, biodegradation, application, environmental pollutants

## Abstract

The treatment of environmental pollution by microorganisms and their enzymes is an innovative and socially acceptable alternative to traditional remediation approaches. Microbial biodegradation is often characterized with high efficiency as this process is catalyzed via degrading enzymes. Various naturally isolated microorganisms were demonstrated to have considerable ability to mitigate many environmental pollutants without external intervention. However, only a small fraction of these strains are studied in detail to reveal the mechanisms at the enzyme level, which strictly limited the enhancement of the degradation efficiency. Accordingly, this review will comprehensively summarize the function of various degrading enzymes with an emphasis on catalytic mechanisms. We also inspect the expanded applications of these pollutant-degrading enzymes in industrial processes. An in-depth understanding of the catalytic mechanism of enzymes will be beneficial for exploring and exploiting more degrading enzyme resources and thus ameliorate concerns associated with the ineffective biodegradation of recalcitrant and xenobiotic contaminants with the help of gene-editing technology and synthetic biology.

## 1. Introduction

The burden of disease and death attributable to environmental contaminants is becoming a public health challenge worldwide [1]. In the last few decades, the environment is being continuously polluted by a large array of pollutants with different structures and ecotoxicities. These pollutants are released from several anthropogenic sources and pose a major threat to ecosystem security and public health, including cancer, type 2 diabetes, and damage to the immune system [2]. Environmental pollutants are commonly divided into organic pollutants and inorganic pollutants. Organic pollutants are widely distributed in the environment, including pesticides, polycyclic aromatic hydrocarbons (PAHs), polychlorinated biphenyls (PCBs), etc. Of particular concern are the persistent organic pollutants (POPs), such as aldrin, DDT, chlordane, and hexachlorobenzene. These POPs are extremely harmful to public health due to their intrinsic chemical stability, recalcitrance, acute toxicity, and mutagenicity or carcinogenicity [3]. Due to the acceleration of global urbanization, modern industries synthesize a huge amount of POPs that are released into the environment [4].

Despite the threat caused by POPs, agricultural activities remain one of the main sources of environmental pollutants. During the traditional agricultural process, excessive use of pesticides has led to continuous contamination of the environment. These pollutants are poorly degraded through natural microbes and plants, and hence, persist in the environment for decades [5]. Moreover, they can spread to great distances by wind and ocean current, including the polar regions and the open oceans [6]. There is concern about the presence of pollutants in the environment because of their long-term residual, bioaccumulative, and toxic nature. Therefore, the study of new and effective degradation mechanisms for pollutants has become an international focal point.

**Figure 1:**
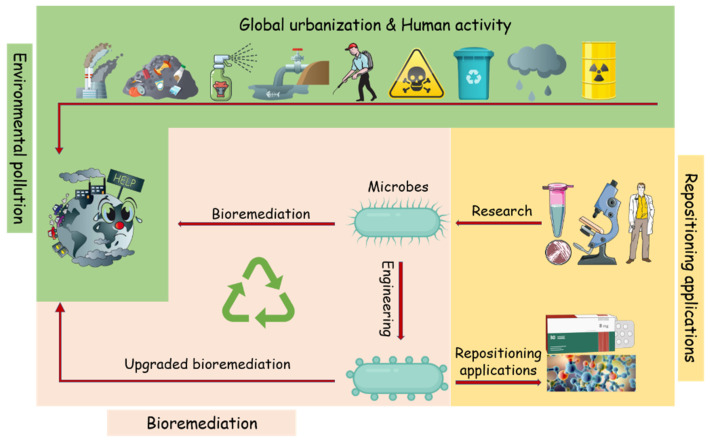
Schematic illustration of the microbial bioremediation of environmental pollutants and its “expanded” applications. Emerging contaminants are often released into the environment, causing harmful impacts and health problems. Researches on the mechanism of the pollutants bioremediation microbes and enzymes will help the application of bioremediation to environmental clean-ups, and the characterize enzymes will also contribute to industrial processes such as pharmaceutical and chemical compounds.

Bioremediation was viewed as the safest and eco-friendly procedure to combat anthropogenic compounds in ecosystems. Many microbes and microbial enzymes with potential bioremediation abilities have been isolated and characterized [7,8]. However, in some cases, they are unable to degrade the targeted pollutant completely or are ineffective in the real natural environment. Nevertheless, with the enhanced understanding of the catalysis mechanisms involved in the degradation of pollutants, the application of these degradation enzymes has aroused extensive attention. Many pollutants degrading enzymes have been applied in industry and modern agriculture, such as biosensors and drug synthesis [9,10] (Figure 1). This study will present a comprehensive overview of the latest research progress in the field of pollutant treatment and degradation and mainly focus on pollutant-degrading enzymes and their industrial applications.

## 2. Enzymes Involved in Organic Pollutant Degradation and Transformation

### 2.1. Oxygenases in Degradation of Aromatic Compounds

Monooxygenases and dioxygenases are the two main subgroups of oxygenases. They are involved in the process of desulfurization, dehalogenation, the oxidative release of the nitro group, and hydroxylation of various aromatic and aliphatic compounds [11]. Alkane monooxygenases are one of the best-studied monooxygenases, which catalyze the first step of alkane degradation. Two common alkane monooxygenases are AlkB-related monooxygenases and the cytochrome P450 family proteins [12]. These two categories of enzymes can utilize short-chain alkanes for growth. Furthermore, flavin-binding and thermophilic soluble long-chain alkane monooxygenase AlmA and LadA have also been found to be involved in the hydroxylation of long-chain alkanes [13,14]. Interestingly, several bacteria are found to contain multiple alkane monooxygenases [15,16]. These coexistent of multiple alkane hydroxylases were considered to complement and expand the range of substrates, thereby enhancing the utilization ability of microbes in some specific environments [17].

In addition to alkane monooxygenases, various monooxygenases have been characterized with distinct functions in environmental pollutant degradation recently. A new study identified and characterized the conserved *bapA* gene in *Aspergillus* that encodes a P450 monooxygenase was necessary for the metabolic utilization of Benzo (α) pyrene (BaP), the main component of PAHs [18]. Besides, the para-nitrophenol 4-monooxygenase (PnpA), involved in the p-nitrophenol (PNP) catabolic pathway of the *Pseudomonas* sp., converts PNP to para-benzoquinone by denitration [19].

**Figure 2:**
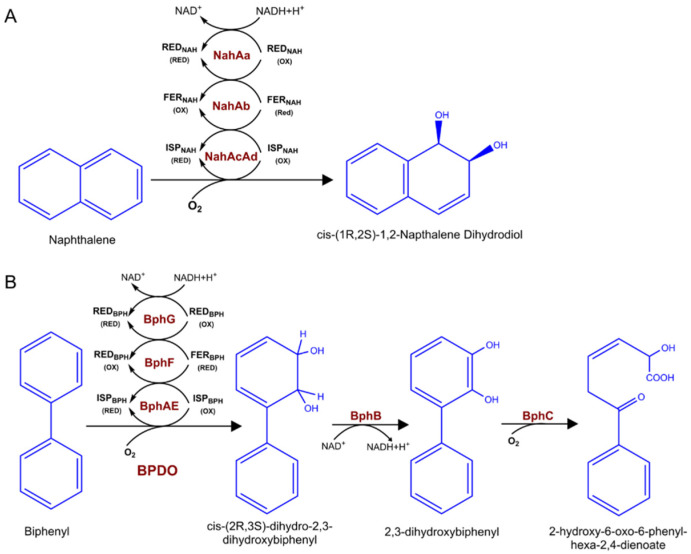
Naphthalene and biphenyl catabolic pathway enzymes and metabolites. (**A**) Naphthalene catalyzed by three-component NDO to cis-(1R,2S)-1,2-napthalene dihydrodiol. (**B**) The four enzymatic steps of the bacterial biphenyl metabolic pathway catalysis by BPDO. red, reduction; ox, oxidation.

The widely studied dioxygenases that hydroxylate aromatic hydrocarbons, playing a key role in the biodegradation of various environmental pollutants. The major aromatic pollutants include PAHs and PCBs. For PAHs, the aerobic metabolism initiates via oxidation of the benzene ring through dioxygenase enzymes by introducing two hydroxyl groups to aromatic compounds. Take naphthalene as an example, there is a large diversity of bacteria that can oxidize naphthalene using naphthalene dioxygenase (NDO) enzymes (Figure 2A), including organisms from the genus *Pseudomonas*, *Rhodococcus*, as well as *Mycobacterium* [20,21]. Common NDOs are multicopper enzymes, composed of an electron transport chain and a terminal oxygenase [22]. Biphenyl is another common widespread organic pollutant belonging to PAHs. Biphenyl utilizing bacteria catabolize biphenyl as well as some PCBs into chlorobenzoic acids by using biphenyl dioxygenase (BPDO) via an oxidative route [23]. Similar to NDO enzymes, BPDO is made up of three components (Figure 2B). The catalytic component (BphAE), ferredoxin (BphF), and ferredoxin reductase (BphG), the latter two components serve as electron transfer from NADH to BphAE [24,25].

### 2.2. Laccases Involved in the Ring Cleavage of Aromatic Compounds

Laccases are broad-spectrum biocatalysts for the degradation of many phenolic compounds, including phenols, polyphenols, and PAHs, which can be easily detected in wastewaters from industries and hospitals [26]. Generally, PAHs are hard to be removed by microbes due to their high hydrophobicity. However, laccases can be used to catalyze the oxidation of PAHs with the assistance of some redox mediators. Within the catalyzation process, aryl radicals are derived from PAHs, followed by the generation of quinones. Prior publications found that the catalysis activity of laccases can be significantly improved via adding extra copper during PAHs oxidation process. However, Zeng et al. found that bacterial laccase CotA from *Bacillus subtilis* can oxidize PAHs in a copper-independent way with higher laccase activity, indicating that CotA could be regarded as a promising candidate for PAH remediation [27].

Laccases have confirmed their ability in the degradation of phenolic compounds. Phenolic compounds can directly oxidize via laccase resulting in the formation of phenoxy radicals, and subsequently undergo phenolic coupling or oxidative coupling via either C-O or C-C bonds [28]. It is notable that reaction products produced via laccase-catalyzed oxidative coupling are usually non-soluble and can be easily separated by sedimentation, several of them of polymeric nature [29]. Overall, laccases initiate the oxidation and polymerization of phenolic compounds to form inert, nontoxic polymers represent a promising environmentally-friendly technology in bioremediation. These mechanisms behind the degradation process, such as phenoxyl-radical mediated coupling, can be applied to phenolic compounds elimination.

### 2.3. Hydrolytic Lipases/Esterases Involved in Bioremediation

Hydrolysis is an important approach in the detoxification of contaminants. Hydrolytic enzymes such as esterases and lipases can split the ester bond of recalcitrant pollutants to relieve their toxicity. This feature enables lipases and esterases promising prospects for the biodegradation of plastic waste, organophosphate, and pesticides. Aryloxyphenoxy propionate (AOPP) herbicide is a class of highly effective herbicide used in agriculture, including fenoxaprop-ethyl (FE), cyhalofop-butyl (CB), haloxyfop-R-methyl (HM), quizalofop-p-ethyl (QE), and clodinafop-propargyl (CP). The FE hydrolase Feh from Rhodoccocus catalyzes the first step of FE biodegradation, which converts fenoxaprop-ethyl to fenoxaprop acid by cleavage of the ester bond [30]. Feh can also convert CB, HM, and QE to their corresponding acids. ChbH is another esterase responsible for the hydrolysis of cyhalofop-butyl (CB) to cyhalofop acid (CA) in *Pseudomonas azotoformans* QDZ-1 [31]. In the case of amide herbicides, arylamidase AmpA, purified from *Paracoccus* sp. FLN-7, catalyzes the amido bond cleavage of amide herbicides, such as propanil, propham, and chlorpropham [32]. Pyrethroids are a class of insecticides used worldwide that demonstrates low mammalian toxicity and high effectiveness, which are commonly used around the home and in agricultural production to control insects. Thus far, various pyrethroid-degrading enzymes have been cloned and characterized to be capable of transforming a wide range of pyrethroid pesticides, such as PytY, PytH, EstP, Sys410, and so on. However, none of the reported enzymes can degrade pyrethroids efficiently and stably [33,34,35]. Through random mutagenesis and secretory expression of Sys410, Liu et al. obtained a mutant enzyme with enhanced activity and thermostability, which able to degrade many pyrethroids and exceeding a hydrolysis rate of 98% [36].

Similar to pyrethroid pesticides, organophosphate pesticides (OPs) also contain an ester bond, which forms the bulk of pesticides that accounting for more than 30% of the world pesticides market. Degradation of OPs mainly occurs by the hydrolysis of the phosphorus-ester (P-S) bond. The best described bacterial enzymes for OPs metabolism is the Opd and its homologs, which are primarily classified as phosphotriesterases (PTEs) and belong to the amidohydrolase superfamily. Thus far, several different types of Oph were described by literature, including *opd*, *opdA*, *opdB*, *ophc2*, *hocA*, *adpB*, and so on. Among them, the *opd* gene has been much more studied than the other organophosphorus hydrolase genes, which were first found to be plasmid-encoded from *Sphingobium fuliginis*, *Brevundimonas diminuta* and has rapidly spread to numerous other bacteria [37].

### 2.4. Heavy Metal Transforming Enzymes

Heavy metals are characterized by densities greater than 5 g/cm^3^, which are either of natural origin or result from anthropogenic activities. Mercury (Hg) is one of the highly toxic and widespread heavy metals [38]. Microorganisms have evolved some astonishing arrays of dedicated resistance systems to adapt to mercury-contaminated environments. One of the well-known bacterial mercury resistance systems is a set of operon genes termed the *mer* operon, which reduces ionic Hg (Hg^2+^) to the volatile elemental form (Hg0). In general, the *mer* operon is composed of several linked genes in a cluster that is responsible for the transport and transformation of inorganic and organic mercury. Typical *mer* operon includes the organomercurial lyase (MerB), which performs the demethylation process by split the methyl group to generate methane (CH_4_) and Hg(II), subsequently, another mercuric reductase (MerA) reduces the Hg(II) to the volatile form. Besides, inner membrane-spanning proteins MerT/C/E/F/G are responsible for the transportation of Hg^2+^ to the cytoplasm, where Hg^2+^ was further reduced by MerA. However, this process only happened in some aerobic prokaryotes, such as *Geobacter*, *Staphylococcus*, *Pseudomonas*, etc. [39]. In addition, the initiation of *mer* pathway needs extremely high Hg concentrations (usually, micromolar) [40], which are irrelevant to most natural Hg-contaminated environments, where Hg or CH_3_Hg^+^ concentrations usually range from picomolar to nanomolar [41]. By using *Methylosinus trichosporium* OB3b as a model methanotroph, Lu et al. report a new CH_3_Hg^+^ demethylation pathway by methanotrophs, which could degrade Hg at relatively low concentrations. Methanotrophic-mediated CH_3_Hg^+^ degradation is remarkably different from the classical *mer* pathway, in which CH_3_Hg^+^ was initially bond with methanobactin, followed by cleavage of the C-Hg by methanol dehydrogenases [42].

**Figure 3:**
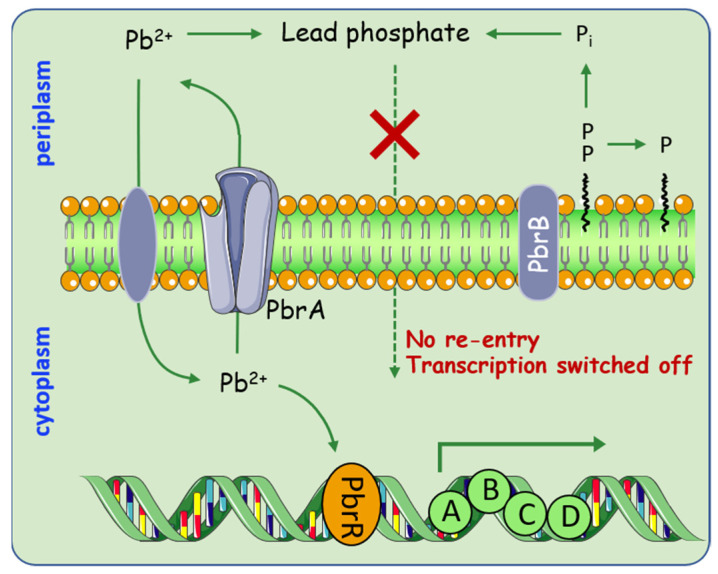
Overview of processes involved in heavy metal metabolism medicated by *pbr* operon. Pb^2+^ enter the cell through specific transporters, which subsequently stimulate transcription of the *pbr* operon. PbrA starts to pump Pb^2+^ to the periplasm, while PbrB dephosphorylates its substrates yielding inorganic phosphate. The process enables the periplasmic free Pb^2+^ sequestered as a phosphate salt.

Apart from Hg pollution, lead (Pb) is considered to be one of the leading pollutants in the environment. This has motivated researchers to explore the diverse mechanisms that microorganisms employ in maintaining resistance to Pb. Special attention is given to the Pbr system, which uses a special strategy by combining efflux and precipitation [43,44]. In general, the *pbr* operons are clustered into two transcription units, *pbrUTR* and *pbrABCD* [45] (Figure 3). This Pb resistance is achieved through the cooperation of the P-type ATPase, PbrA, that non-specifically exports Zn^2+^, Cd^2+^, and Pb^2+^; together with the pyrophosphate phosphatase encoded by PbrB that specifically increases Pb resistance. This new Pb resistance model allows Pb^2+^ removal from the cytoplasm by PbrA; it is then sequestered as a phosphatase salt with the inorganic phosphate produced by PbrB in the periplasm. Although lead efflux is one of the prominent resistance mechanisms in bacteria, intracellular sequestration medicated by metallothionein genes were regarded as another strategy to alleviate lead toxicity. For example, the presence of bacterial metallothionein genes, *bmtA* and *smtAB* [46,47]. Sharma et al. found that BmtA from *Providencia vermicola* can sequestrate 155.12 mg/g of lead in the periplasmic space by converting them into lead sulfite [48].

**Figure 4:**
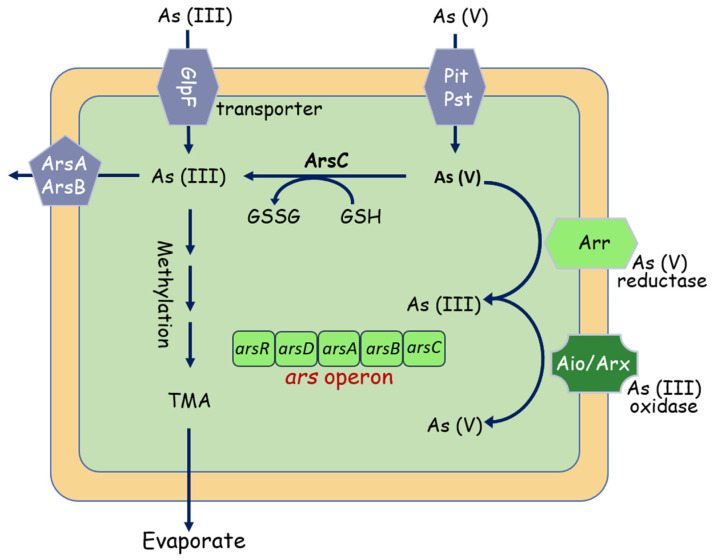
Overview of bacterial interactions with arsenic. Under aerobic conditions, As(V) enters the cell via phosphate uptake systems (PstA, PstB). As(V) is then reduced by the arsenate reductase ArsC to As(III). As(III) can also directly be taken up aquaglyceroporins such as GlpF, and then bound by the As(III)-binding chaperone ArsD and extruded by ArsAB efflux pump. As(III) can also be methylated, forming the trimethyl arsine (TMA), pumped out by the ArsP transporter.

More notably, arsenic (As) pollution has attracted increasing attention due to its carcinogenic toxicity [49]. Arsenate (AsV) and arsenite (AsIII) are the two dominant inorganic species [50,51], AsIII is more toxic and mobile than AsV. To cope with As toxicity, microbes have evolved multiple mechanisms, in which the *ars*, *aio*, and *arr* operons are considered to be the extensively studied pathways involved in As metabolism [52]. Starting with the *ars* system (Figure 4), the *ars* operon is a detoxification system that reduces AsV to AsIII, the *asrC* gene codes an arsenate reductase for the reduction of As(V) to As(III), and the AsrB functions as an As(III) expulsion pump, facilitating its cellular extrusion [53,54,55]. The *aio* and *arr* systems are demonstrated able to use arsenic during its metabolism activities. The arsenite oxidase (Aio) system can take electrons from arsenite and oxidizing them to arsenate, and the arsenate respiratory reductase (Arr) system catalyzes the reduction of arsenate to arsenite at the end of the respiratory chain. [56,57]. Indeed, thanks to the presence of *aio* operon, As(III) is oxidized in the periplasm by arsenite oxidases (AioAB) into the less bioavailable form As(V) [58].

## 3. Expanded Application of the Pollutant-Degrading Enzymes in Industries

### 3.1. Application of the Pollutant-Degrading Enzymes in Biosensors

**Figure 5:**
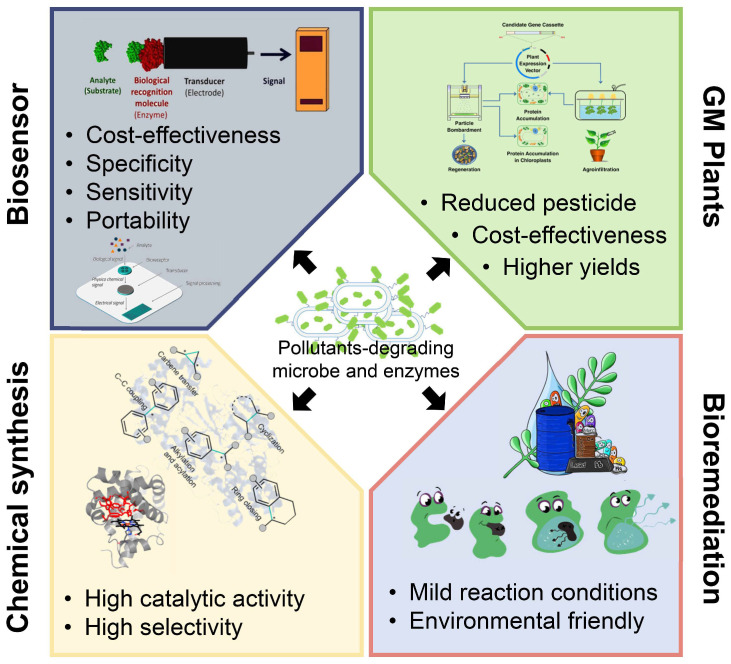
The figure summarizes major applications of pollutants-degrading microbes and enzymes and the representative features of each one. Pollutants-degrading microbes and enzymes are widely used in biosensors, GM pants, chemical synthesis, and bioremediations. Many of these applications show prominent advantages such as cost-effectiveness, high selectivity, environmental-friendly, etc.

Biosensors are analytical devices composed of a biorecognition sensing element integrated with different physicochemical transducer systems [59]. Various enzymes, antibodies, and microorganisms have been applied in the construction of biosensors [60]. Recently, many pollutant-degrading enzymes have been successfully used to develop biosensors (Figure 5) due to their sensitivity, specificity, portability, cost-effectiveness, and the possibilities of their miniaturization [61].

One successful attempt at the application of biosensors in environmental pollutant detection is that of the synthetic organophosphorus (OPs) biosensors. The most extensively reported enzymes were acetylcholinesterase (AChE) and butyryl-cholinesterase (BChE), which were widely regarded as the initial enzymatic sensors for the detection of organophosphates. The enzyme-catalyzed hydrolysis of the choline esters results in a decrease in the amount of acid released, and hence, causing a shift in the pH, which was further detected by the AchE biosensors [62]. The amperometric AChE biosensors function via detecting the concentration of thiocholine, a hydrolysate of acetylthiocholine. The existence of OPs inhibited the hydrolytic activity of AChE, and therefore, less thiocholine is produced [63]. However, these biosensors often lack selectivity because they are easily interfered with by cholinesterase inhibitors, such as hypochlorite, detergents, carbamates, heavy metals, fluoride, and nicotine [64]. To avoid this disadvantage of AChE-based biosensors, several OP-degrading enzymes were identified and successfully used to develop for the detection of OPs directly. For instance, OPH and organophosphorus acid anhydrolase (OPAA) show considerable potential in OP biosensor applications [65].

Many chromophoric compounds are not visible to the naked eyes, but their absorbance can be easily detected at definite wavelengths (λ) using a spectrophotometer. For this purpose, spectrophotometers are commonly combined in the construction of colorimetric biosensors. *Flavobacterium* sp. cells were entrapped in a glass fiber filter and used to detect the methyl parathion pesticide [66]. This biosensor was based on the enzyme’s activity of OPH in *Flavobacterium* sp., which hydrolyzed methyl parathion into PNP, further measured by its absorbance at 400 nm. This colorimetric biosensor can detect methyl parathion at extremely low concentrations (0.3 µM), which was much less than that obtained by amperometric biosensors [66]. Another optical biosensor via immobilizing *Pseudomonas* sp. P2 cells on silica for the detection of polychlorinated biphenyls. The detection was based on the yellowish metabolic products with a characteristic wavelength at 398 nm [67]. In addition, an amperometric enzyme biosensor based on parathion hydrolase was used for monitoring parathion. These enzymes catalyze the hydrolysis of parathion to form p-nitrophenol, which was detected by its anodic oxidation [68].

### 3.2. Application of Pollutant-Degrading Enzymes in the Syntheses of Pharmaceutical Precursors

Given the increasing environmental and economic pressure to use renewable sources of energy and chemical feedstock in industry, the use of enzymes as biocatalysts has enormous potential in the industrial manufacture of fine chemicals and pharmaceuticals [69]. Many intermediate products produced by microorganisms during the metabolic process are value-added substrates for pharmaceutical production. For example, carbazole is a tricyclic aromatic N-heteroatomic compound, which also functions as a well-known pollutant due to coal gasification. Carbazole is a kind of chemical feedstock for the production of dyes, reagents, insecticides. However, the biotransformation of carbazole to hydroxy-carbazole is of great interest because hydroxylated carbazole derivatives are value-added substances in the pharmaceutical industry [70]. Bacterial dioxygenases, such as biphenyl dioxygenase and naphthalene 1,2-dioxygenase often catalyze the oxidation of aromatic hydrocarbons and related heterocycles, including carbazole [71].

During nicotine degradation, a high valuable intermediate, named 6-Hydroxy-3-succinoyl-pyridine (HSP) will be produced, which is also an important precursor for the synthesis of drugs and analgesic compounds [72]. However, modern organic chemistry is unable to synthesis the HSP. To overcome this predicament, the biotransformation of HSP from nicotine becomes the only operative method. Over the last decade, *Pseudomonas putida* S16 has been used as a model strain to investigated the pyrrolidine pathway of nicotine degradation. In some prior published studies, the whole cells of *P. putida* S16 produced a low concentration of HSP [73]. Later, nicotine degradation genes belong to the nic2 operon were identified, and the enzymes responsible for the transformation of 3-succinoyl-pyridine (SP) into HSP were also identified. HSP was later transformed into 2,5-dihydroxy-pyridine (2,5-DHP) by HspB [74]. Based on these findings, a genetically constructed strain *P. putida* P-HSP based on the inactivation of the HspB in S16. Thus, it realized a 3.7-fold higher production of HSP than the non-engineered strain S16 [75]. Interestingly, Hu and colleagues demonstrated that HSP could directly bind to NicR2 to block the binding of NicR2 to the *spm* promoters, thus initiate the transcription of *spmABC* to catalyze SP into HSP [76].

### 3.3. Application of Pollutant-Degrading Enzymes in the Biocatalysts of Chemical Products

The ability of microorganisms, particularly some pollutant-degrading microbes, to metabolize a wide variety of organic compounds has stimulated interest in exploring biotechnological routes for the production of useful chemicals (Figure 5, Table 1). The selective oxygenation of aromatic compounds catalyzed by oxidoreductases has been widely concerned, and various products have been synthesized via these enzymes. Two classes of compounds that are of interest are the cis-dihydrodiols and catechol. Cis-dihydrodiols are formed by the deoxygenation of aromatic rings and may be useful educts for the production of synthetic polymers; catechol is formed by the dehydrogenation of cis-dihydrodiols [77]. Many dioxygenases, monooxygenases, and oxidases have been engineered for the synthesis of catechols, cis-dihydrodiols, and other oxygenated products [78].

Rieske oxygenase (RO) system is composed of several non-heme iron oxygenases, which could catalyze the oxidation of substrates. The RO system is characterized by a high degree of stereo-, regio-, and enantio-specificity. These superiorities have made ROs a desirable platform in biosynthesis [79]. The first step of RO system is the catalyzation of aromatic compounds and the formation of cis-dihydrodiols from aromatic substrates. Toluene dioxygenase (TDO) is one of the most widely studied RO systems, which catalyze a broad range of mono-substituted arenes [80]. As a rough estimate, more than three hundred 2,3-cis-dihydrodiol derivatives have been synthesized using recombinant strains expressing TDO [81].

Despite the synthesis of cis-dihydrodiols, several monooxygenases and dioxygenases which intrinsically catalyze hydrocarbons have been endowed with new functions for oxidizing indole into indigo. Indole is a common nitrogen-containing aromatic pollutant in coking wastewater; however, it can be used for the production of indigo through biotransformation. Besides, many oxygenases have been reported to be able to oxidize indole, including cytochrome P450, naphthalene dioxygenase (NDO), monooxygenase, and flavin monooxygenase (FMO). For example, NDO genes from *Comamonas* sp. MQ was heterogenous expressed in *E. coli*, resulting in a high rate of conversion towards indole and its derivatives [82].

**Table 1:** A summary of recently reported microbes and enzymes employed in industrial synthesis.

Microorganism	Enzyme Activity	Relevant Industrial Synthons	Reference
*Sphingobium yanoikuyae* B1	Rieske oxygenase (RO)	*cis*-dihydrodiols	[83]
*Burkholderia cepacia* G4	Toluene ortho-monooxygenase (TOM)	isoindigo indigo, indirubin, and isatin	[84]
*Pseudomonas putida* UV4	Toluene dioxygenase (TDO)	2,3-*cis*-dihydrodiol Toluene Chlorobenzene Bromobenzene Naphthalene	[85]
*Escherichia coli* BW25113	TDO	1,2-*cis*-dihydrocatechol	[86]
*Sphingomonas* sp. CHY-1	Naphthalene dioxygenase (NDO)	Naphthalene	[87]
*Pseudomonas* sp. NCIB 9816–4	NDO	(R)-1,2-phenylethanediol	[88]
*Pseudomonas* sp. species	Dihydrocatechol dehydrogenase (DHCD)	2,3-Substituted catechols	[89]
*Pseudomonas mendocina* KR1	Toluene-4-monooxygenase (T4MO)	4-Substituted Phenol 3,4-Substituted Catechol	[90]
*Pseudomonas putida* S12	TOM-Green	1-Naphthol	[91]
*Escherichia coli* TG1	Toluene-4-monooxygenase (T4MO)	Phenol 2-Naphthol	[92]
*Pseudomonas putida* S12	Styrene monooxygenase (SMO)	Styrene oxide	[93]
*Pseudomonas putida* KT2440	SMO	Epoxide	[94]
*Rhodococcus* sp. DK17	o-xylene dioxygenase	3-methylbenzylalcohol and 2,4-dimethylphenol	[95]
*Pseudomonas *putida** KT2440	ω-transaminases	2-hydroxy ketone	[96]

## 4. Conclusions and Perspectives

The dissipation of recalcitrant residual pollutants in the environment is influenced by diverse biological processes, including microbial biodegradation, biosorption, phytoremediation, and so on. In recent times, a growing concern of the harmful effects of environmental contaminants has led to a marked increase in research into various strategies that can be adopted to clean up the contaminated environment. We are just beginning to understand and thus, fully exploit the natural resources for bioremediation. At the present stage, we can describe novel strains, enzymes, and metabolic routes involved in bacterial-mediated pollutant degradation.

The rise of new biotechnologies in the last decade has enabled to unlock the functional potential of microbial-assist bioremediation. Many microorganisms with remarkable catabolic potential, especially those originating from highly polluted environments, have been isolated and characterized. In particular, many enzymes, especially produced by those uncultivable bacteria, have been discovered thanks to the omics approach. Currently, multiple advanced approaches, including metagenomics, proteomics, transcriptomics, and metabolomics, are successfully employed for the characterization of pollutant-degrading microorganisms, novel proteins, and catabolic genes involved in the degradation process. These revolutionary advanced molecular practices deliver deeper insights into microbial activities concerning their enzymes and metabolic pathways, which also contributed to finding biocatalysts that are naturally adapted to industrial constraints.

In addition to their intrinsic degrading ability, many pollutants degrading enzymes possess special functions and great application prospects in biocatalysis. Thus, many microbes and the related degrading enzymes have been successfully adapted in diverse areas, such as in the preparation of industrial biosensors, intermediates of pharmaceutical progress, medical bioremediation, etc. (Figure 5). These applications not only promote the sustainable utilization of biological resources but also yield enormous economic benefits and huge social effects. However, only a small fraction of these pollutants degrading microbes and their enzymes are recognized and applied.

Through evolution, nature has fashioned a plethora of enzymes to catalyze diverse reactivities that make life possible, such as the pollutant degrading enzymes. However, most of these natural pollutant degrading enzymes are often characterized by low efficiency and stability, thereby limiting the implementation of bioremediation and other industrial processes [97]. Natural enzymes often need to be improved by protein engineering to optimize their function in non-native environments. Recent technological advances have greatly facilitated this process by providing the experimental approaches of directed evolution or by enabling computer-assisted applications [98]. Computational techniques can be used to engineer enzymatic reactivity, substrate specificity and ligand binding, access pathways and ligand transport, and global properties such as protein stability, solubility, and flexibility. Combining in silico design with high-throughput screening for the creation of hyperpower pollutant degrading enzyme is much to be expected from computational enzyme design.

With the development of revolutionary biotechnologies, we can discover more prominent microorganisms and enzymes in bioremediation. Depending on these degrading microorganisms and enzymes, the bioremediation of pollutants could be a promising environmentally friendly and effective way to remove them from the environment. Moreover, to achieve spectacular breakthroughs in the application of bioremediation, more studies are needed to understand the interaction between environmental pollutants and organisms and on the fate, survival, and activities in real natural conditions to intersect with systems biology and metabolic engineering approach. Such as the designing of artificial microbial consortia will provide the ground for successful interventions into environmental processes and thereby lead to optimized strategies for bioremediation.

## Data Availability

No new data were created or analyzed in this study. Data sharing is not applicable to this article.
